# Putting scales into evolutionary time: the divergence of major scale insect lineages (Hemiptera) predates the radiation of modern angiosperm hosts

**DOI:** 10.1038/srep23487

**Published:** 2016-03-22

**Authors:** Isabelle M. Vea, David A. Grimaldi

**Affiliations:** 1 Richard Gilder Graduate School, American Museum of Natural History, Central Park West at 79th street, New York, NY 10024, USA; 2 Division of Invertebrate Zoology, American Museum of Natural History, Central Park West at 79th street, New York, NY 10024, USA

## Abstract

The radiation of flowering plants in the mid-Cretaceous transformed landscapes and is widely believed to have fuelled the radiations of major groups of phytophagous insects. An excellent group to test this assertion is the scale insects (Coccomorpha: Hemiptera), with some 8,000 described Recent species and probably the most diverse fossil record of any phytophagous insect group preserved in amber. We used here a total-evidence approach (by tip-dating) employing 174 morphological characters of 73 Recent and 43 fossil taxa (48 families) and DNA sequences of three gene regions, to obtain divergence time estimates and compare the chronology of the most diverse lineage of scale insects, the neococcoid families, with the timing of the main angiosperm radiation. An estimated origin of the Coccomorpha occurred at the beginning of the Triassic, about 245 Ma [228–273], and of the neococcoids 60 million years later [210–165 Ma]. A total-evidence approach allows the integration of extinct scale insects into a phylogenetic framework, resulting in slightly younger median estimates than analyses using Recent taxa, calibrated with fossil ages only. From these estimates, we hypothesise that most major lineages of coccoids shifted from gymnosperms onto angiosperms when the latter became diverse and abundant in the mid- to Late Cretaceous.

Living insect species that feed on vascular plants comprise some 40% of the described insect diversity^1^, and so it appears that plants have had a profound effect on the diversification of insects. In comparisons between multiple sister-pairs of insect groups, for example, where one group is herbivorous and the other not, the former was found to be almost always far more diverse^2^. Moreover, within major groups of herbivorous insects the great proportions of species feed on angiosperms, the sister lineages having just a few species that feed primarily on gymnosperms or plant detritus. Good examples include the hyperdiverse weevils (Coleoptera: Curculionoidea)^3^ and the Lepidoptera^1^,^4^, the latter the largest lineage of plant-feeding animals. Since both of these insect groups are known to pre-date the first fossil angiosperms, and well preceded the angiosperm radiations in the mid-Cretaceous, it has commonly been inferred that the “colonization” or shift to angiosperms promoted insect diversification. Perhaps no other group has been a more popular subject for this topic than butterflies (Lepidoptera: Papilionoidea), in which diversification of the whole group and component lineages have been tied to the diversification of various host plant lineages^5^,^6^,^7^,^8^.

Without question, the pervasiveness of insect herbivory has been a major selection pressure on plants, which evolved pharmacopias of toxic secondary metabolites to defend against the insects^5^,^9^, and insects in turn evolved resistance to the toxins^7^. However, the role that herbivory *per se* has played in insect diversification, speciation, or cladogenesis, is unclear. First, the 280,000 known angiosperms^10^ constitute the largest majority of the living vascular plant diversity, so a preponderance of insect herbivores on angiosperms would be expected based on chance alone. Second, there was no apparent increase in the number of insect families during the Cretaceous when angiosperms radiated^11^. Third, there are very few definitive examples with appreciable correspondence in insect-host plant relationships^12^, exceptions being groups like fig wasps (Hymenoptera: Agaonidae)^13^ and yucca moths (Lepidoptera: Prodoxidae), whose larvae feed on the same hosts for which the adult insects are also specialized pollinators^14^. Fourth, a few studies done to date indicate that some lineages of herbivorous insects actually diversified after their host taxa^15^. Lastly, and which would explain the general lack of co-speciation between insects and their host plants, a population-genetic mechanism for how host-plant use would lead to sympatric divergence has been empirically controversial and largely unproven^16^,^17^. We explored the diversification of a major group of phytophagous insects, the scale insects (Hemiptera: Coccomorpha), of which 97% of the living species feed on angiosperms, and the group as a whole has a superb fossil record for the past 130 million years.

Coccomorpha are Hemiptera, all of which possess mouthparts modified into a rostrum, comprised of highly specialized mouthpart appendages that allow them to pierce and siphon liquids, from plant vascular fluids to insect hemolymph and vertebrate blood. This feature not only adapted most hemipterans to an exclusive diet of plant fluids (90% of them^1^), but as a consequence some members have developed intimate symbiotic relationships, such as with ants, which feed on their excreted honeydew^18^ and with endosymbionts that nutritionally supplement a diet of plant fluid^19^. Within the strictly phytophagous suborder Sternorrhyncha (also including whiteflies, plant lice and aphids), the most speciose infraorder is Coccomorpha^20^ (scale insects and mealybugs), representing half of the species diversity and accounting for some of the most important plant pests. There are some 8,000 described species of Coccomorpha^21^, with some 52 families (33 Recent and 19 extinct); the sister group, Aphidomorpha, comprises three Recent families with about 4,500 species^22^. Despite the impact of scale insects on agriculture and an extensive body of taxonomic work, very few studies have addressed their evolutionary history, which clearly impedes evolutionary understanding of relationships to their host plants. Higher-level phylogenetic relationships are gradually becoming better resolved^23^,^24^, diverse new fossils are being uncovered^25^,^26^,^27^,^28^, allowing timelines of lineage divergence to be assessed. The recognized monophyletic lineage neococcoids^24^,^29^ constitutes 90% of Coccomorpha Recent species (e.g., Pseudococcidae (Fig. 1A), Coccidae (Fig. 1B), and Diaspididae) and comprises half of the families. In contrast, the remaining scale insect families, comprising the informal, basal paraphyletic grade “archeococcoids” (Fig. 1C), are significantly less diverse today, with only 10% of the species.

How did the neococcoids become so diverse today? Most scale insects feed on angiosperms^24^, with some exceptions amongst the archeococcoids, such as the Matsucoccidae (ca. 30 Recent species, exclusively feeding on conifers), or the Ortheziidae (ca. 200 Recent species, mostly found in leaf litter or on lichens and mosses^30^). Thus, the straightforward hypothesis is that neococcoid families originated and diversified as a consequence of the angiosperm radiations in the mid-Cretaceous^31^,^32^. Scale insects have an exemplary fossil record for the past 130 Ma, since they are one of the most abundant and diverse groups preserved in amber deposits around the world^25^,^26^,^27^, from the Early Cretaceous to the Miocene (ca. 130–20 Ma). In Turonian-aged (90 Ma) amber from New Jersey, for example, Coccomorpha is the most abundant family-level group of insects^33^. Moreover, the microscopic fidelity of preservation in amber allows rigorous phylogenetic interpretation of the inclusions, particularly of such minute insects (Fig. 1D,E). Based on recent discoveries of new coccoids in Cretaceous amber and their phylogenetic relationships^28^, which we further explore here, the view of a Cretaceous radiation of Coccomorpha and Tertiary radiation of neococcoids needs to be revised.

Because of extreme sexual dimorphism in coccoids (Fig. 1A–C), the conspicuous, feeding, and longer-lived adult females are used in taxonomy. In contrast, the ephemeral, winged males are devoid of mouthparts and emerge for reproduction exclusively. For a large majority of coccoid genera and species the male is unknown. Although not an issue for species identification and delineation, this is problematic for phylogenetic studies since the highly reduced, paedomorphic females dramatically differ among families (e.g., Fig. 1A–C). Homologies are obscured, resulting in little phylogenetic study of female morphology and a reliance on molecular data. Although adult male morphological characters are generally more informative phylogenetically than those of females, especially at the family level^29^,^34^, taxon sampling for males remains sparse for many genera and even some families. Nonetheless, fossils are largely based on adult males that wafted into ancient resins^35^, precluding comparison between extinct and Recent taxa based just on taxonomic studies using female morphology. Fortunately, the last decade has been quite fruitful in our knowledge of male morphology, especially for undersampled families^36^,^37^,^38^, and most families have now at least a few male representatives described.

Considering the situation described above, it becomes possible to delve into the divergence time estimates of scale insect lineages. Previous hypotheses involving a timeline for scale insect phylogenetic relationships have been intuitive. Koteja^35^ proposed a scheme of relationships for Recent and extinct families (summarized in Grimaldi and Engel^1^), and comprehensive phylogenetic studies including fossil taxa have been published only recently, based on male morphology^29^, or on the morphology of both sexes^28^. However, so far no quantitative analysis of divergence times has been made for this important phytophagous group. A time-scaled phylogeny can address evolutionary questions such as how divergence times in scale insects compare to the appearance in the fossil record of their major host plant groups.

For insect groups more generally, divergence-time analyses are now produced using large sets of transcriptomic data, and despite advances in computational bioinformatics, most such studies have been limited with the incorporation of fossils. MrBayes 3.2 started to implement a mathematical formulation of prior models for time trees with fossils (the Independent Gamma Rate (IGR) relaxed-clock model with uniform tree prior^39^), allowing one to assess age estimates of lineages at the same time as accommodating the uncertainty of fossil placements. More recently, the Fossilized Birth-Death (FBD) process^40^, which models varying rates of speciation, extinction, and fossilization, was implemented in MrBayes 3.2.6. combined to diversified sampling of extant taxa, which is suitable for studies of higher-level taxa^41^.

Scale insects are an ideal group to test the incorporation of multiple extinct taxa in these analyses, since the fossil record has both comprehensive taxonomic and geological coverage. Despite the great fossilized bias of male scales, and the tedious study of microscopic inclusions in amber, Coccomorpha is ideal for exploring the diversification of a major group of phytophagous insects. Indeed, it is the most diverse family-level group of phytophagous insects preserved in the amber fossil record. Herein, we use tip-dating and total-evidence (fossil and recent taxa, molecular and morphological data) approaches from a dataset of 43 fossils and 73 Recent representatives, using the FBD model with diversified sampling, to examine the divergence times of major lineages within Coccomorpha. This approach allows the incorporation of morphological characters from extinct species, otherwise not possible in the traditional node-calibrated analyses. We also compare these estimates to a node-dating analysis, which includes only Recent terminals and 12 node calibrations based on the fossils that could be placed within extant taxa.

## Results

### Node-dating analysis

First, we inferred a divergence-time estimate using the node-dating approach with the dataset including only Recent taxa (78 Coccomorpha and 5 Aphidomorpha representatives). This dataset comprised both morphological and molecular characters. The analysis was performed using the IGR relaxed-clock model and 12 age priors following an offset exponential distribution, which were assigned across the phylogeny as node calibrations. The calibrations were based on Aphidomorpha and Coccomorpha fossil records ranging from 250 to 45 million years (Table S3).

**Figure 2:**
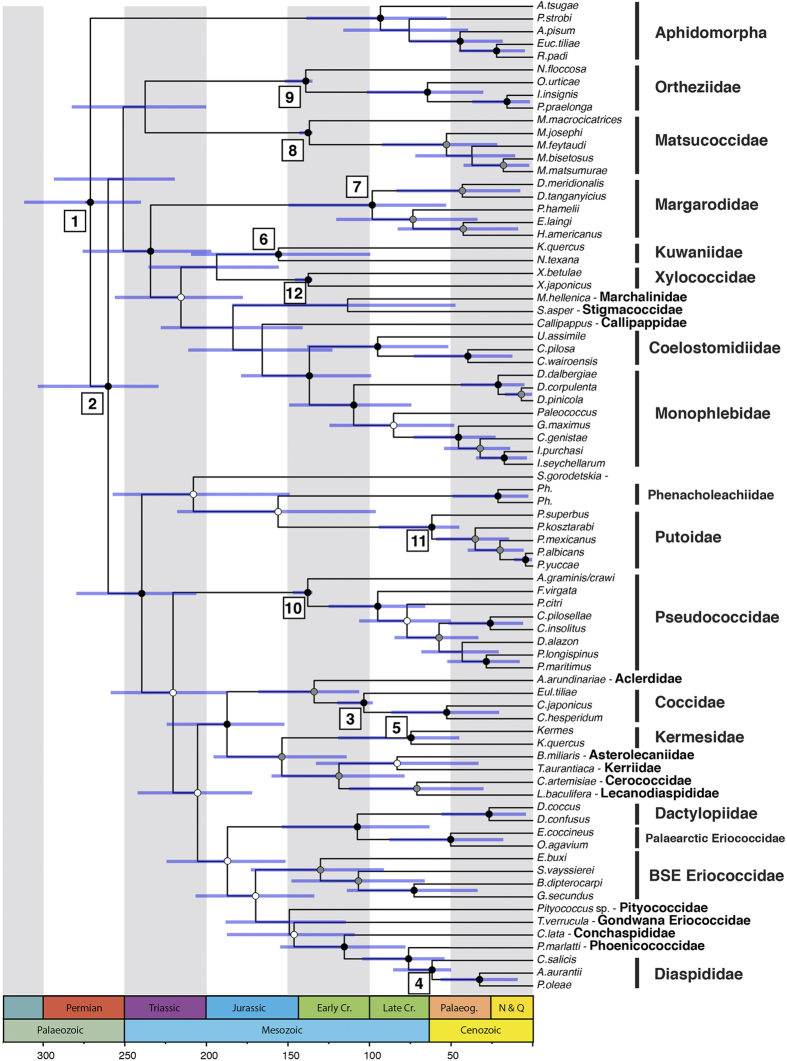
Divergence-time estimates based on a node-calibrated analysis (node-dating) including Recent terminals, using MrBayes 3.2.6, all compatibility summary. Posterior probability: black dot: 95–100%; grey dot: 80–94%; white dot: 50–79%. Node numbers represent each node prior (Supplementary Table S3).

This inference (Fig. 2) resulted in Coccomorpha as a monophyletic group (PP = 100%) that originated 260 [229–303] Ma. All families sampled with more than two representatives were found to be monophyletic lineages (except for Eriococcidae but see^42^). In this analysis, the neococcoids were not monophyletic as it included the archaeococcoid *Pityococcus* sp., found sister to *Tanyscelis, Conchaspis, Phoenicococcus* and *Diaspididae*. The only sampled Recent *Pityococcus* did not include any molecular data and the derived female morphology could have resulted in its unexpected placement. The age of the node including members of the neococcoids and *Pityococcus* was estimated at 220 [187–258] Ma.

Within the infraorder, the analysis reasserted the paraphyly of the archaeococcoid families with regard to neococcoids^24^,^28^,^29^,^34^; with Putoidae, Phenacoleachiidae and Steingeliidae being sister group to the neococcoids and *Pityococcus*. The two lineages (PP > 90%), originated shortly after Coccomorpha (age: 251 [240–312] Ma).

### Tip-dating analysis

**Figure 3:**
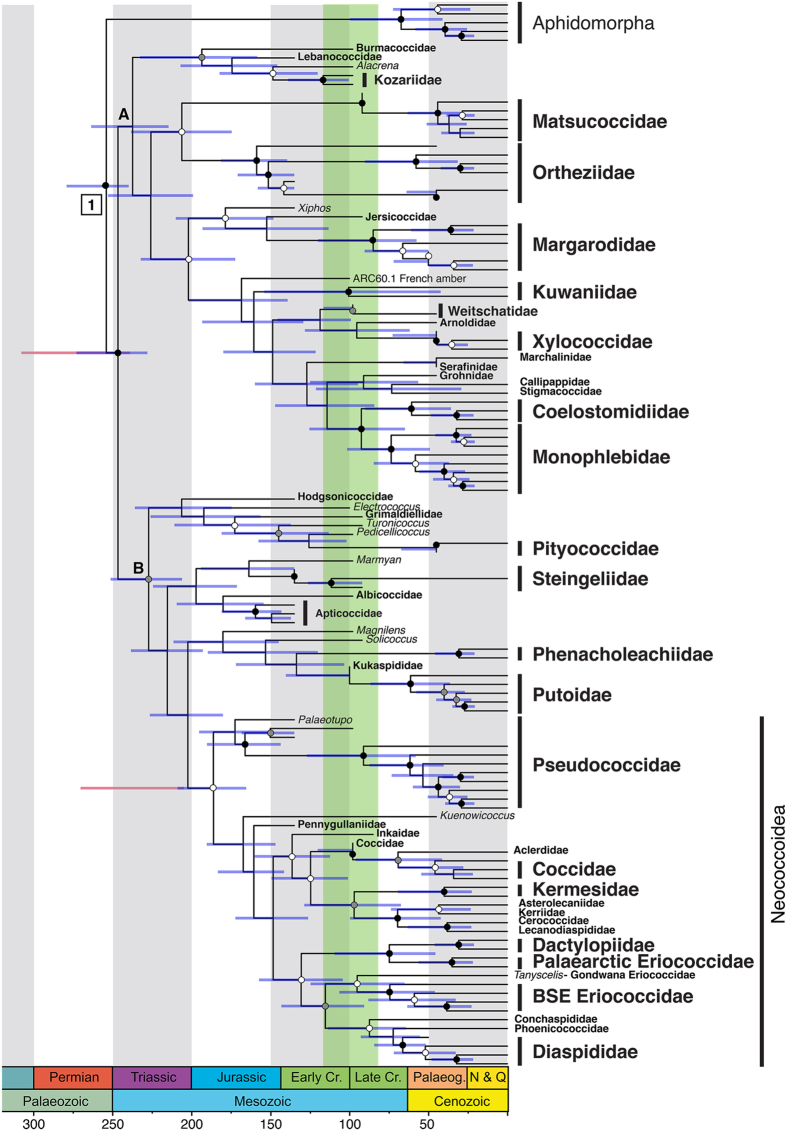
Divergence-time estimates based on a total-evidence approach (tip-dating) including Recent and fossil terminals, using MrBayes 3.2.6, following the Fossilized Birth-Death model, with diversity sample strategy, fixed tip priors, and all-compatibility summary. Posterior probability: black dot: 95–100%; grey dot: 80–94%; white dot: 50–79%. Node number represents root node prior (Table S3). Node letters are clades mentioned in the results. Blue bar: Tip-dating posterior age range using the Fossilized Birth-Death model^40^,^41^ Red bar: Tip-dating posterior age range for Coccomorpha and Neococcoidea lineages using Ronquist *et al*.^39^.

The tip-dating approach age estimates (Fig. 3) were inferred using the IGR relaxed-clock model from 78 Recent and 43 extinct Coccomorpha, as well as five Aphidomorpha outgroups. In this case, fixed age priors were assigned to the tip of each of the 43 fossils (Table S4) and we used the FBD process as the speciation model^40^, coupled with the accommodation of diversified sampling, both recently implemented in MrBayes 3.2.6^41^.

This analysis assessed Coccomorpha as monophyletic (PP = 100%) and originating about 247 [228–273] Ma. Coccomorpha is divided into two main lineages: node A comprises all those families of the parahyletic archaeococcoids whose adult males bear compound eyes (PP < 50%; age 237 [215–264] Ma), with an extinct clade including representatives from the mid- to Early Cretaceous (Burmacoccidae, Lebanococcidae, Kozariidae and *Alacrena*) (PP = 90%; age:194 [158–233] Ma); node B (PP > 70%; age: 227 [206–252] Ma) consists of the monophyletic neococcoid lineage (PP > 50%; age: 186 [166–209] Ma), as well as Putoidae, the relict families Phenacoleachiidae, Pityococcidae, Steingeliidae, and finally seven extinct families (Albicoccidae, Electrococcidae, Hodgsonicoccidae, Kukaspididae, Labiococcidae, Apticoccidae, Grimadiellidae). These families are therefore more closely related to neococcoids than archaeococcoids^43^, even though traditionally classified with the archaeococcoid families.

### Phylogenetic comparison between tip-dating and node-dating approaches

**Figure 4:**
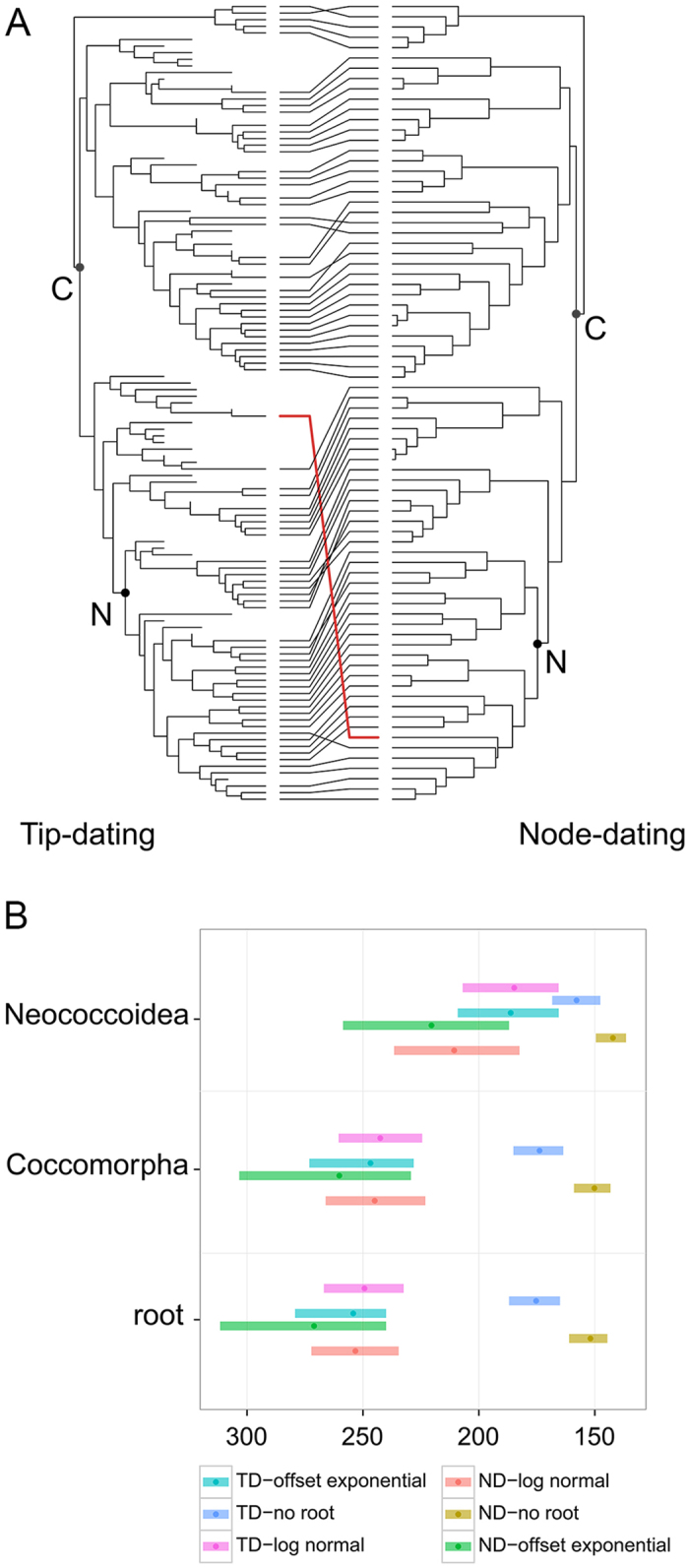
Comparison of the tip-dating and node-dating approaches. (**A**) Face-to-face comparison of the phylogenetic relationships constructed using the R Ape Package. Left: tip-dating, right: node-dating. Red: change in *Pityococcus* placement within the Neococcoidea lineage using the node-dating approach, and outside of the Neococcoidea when fossils are added. C: Coccomorpha, N: Neococcoidea. (**B**) Plot of the posterior ages for the split of Aphidomorpha/Coccomorpha, Coccomorpha and Neococcoidea lineages, obtained from the node-dating and tip-dating approaches with different root priors. The dots represent the median age, and the ranges are the 95% HPD. TD: Tip-dating, ND: Node-dating.

A face-to-face comparison of the topologies obtained from tip- and node-dating approaches revealed a few changes in family relationships (Fig. 4A). First, the major difference appears to be the placement of *Pityococcus*. The node-dating topology found the family to be included in the neococcoid lineage. The incorporation of fossil taxa, including an extinct *Pityococcus* was sampled, rendered the family Pityococcidae included in a group with only fossil representatives (Hogsonicoccidae, *Electrococcus*, Grimadiellidae, *Turonicoccus* and *Pedicellococcus*, Fig. 3), and excluded from the neococcoid lineage, as found in previous studies^29^.

The other families that resulted in different placement from the addition of fossil terminals were the Xylococcidae, Kuwaniidae, Stigmacoccidae and Marchalinidae. The latter two have only one taxon sampled and are families with very low diversity in the present. The Kuwaniidae relationship was moved to become sister group to the lineage including extant Xylococcidae, Marchalinidae, Callipappidae, Stigmacoccidae, Coelostomidiidae and Monophlebidae (Fig. 3), by the addition of several fossils that were hypothesized to be related to the Xylococcidae. The node-dating approach however placed this family as sister to extant Xylococcidae (Fig. 2).

### Effect of priors on age estimates

We performed a series of analyses to test the effect of various priors on the median age estimates and confidence intervals (95% HPD) for the split between Aphidomorpha and Coccomorpha, and the Coccomorpha and Neococcoidea lineages. Figure 4B compares the age estimates of these nodes between the node- and tip-dating approaches and three alternatives of root prior assignment (no root prior, root prior following a log-normal, and offset exponential distribution). In both node- and tip-dating analyses, removing the root prior pushed forward the age estimate of Coccomorpha to about 175 Ma (Fig. 4B and Fig. S4). The two other nodes were also significantly younger. These two approaches without a root prior resulted in younger node estimates. When we assigned a root prior, using an offset exponential distribution in the node dating analysis, we obtained slightly older median ages with wider 95% HPD, a result not found when using the tip-dating approach.

**Figure 5:**
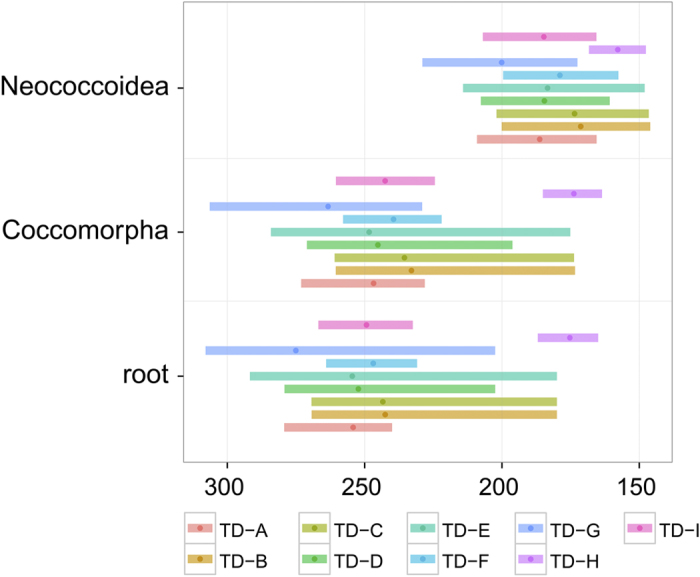
Effects of parameters used in the tip-dating approach. Plot of the posterior ages for the split of Aphidomorpha/Coccomorpha, Coccomorpha and Neococcoidea lineages. The dots represent the median age, and the ranges are the 95% HPD. For a detail on parameters used for each tip-dating analysis, refer to Table 1.

Second, we performed tip-dating analyses to test the effect of tip-prior distributions (assigning a fixed prior age or a uniform prior age to fossil terminals), branch-length priors (uniform tree prior^39^ or FBD model^40^), sample strategy (diversified or ‘fossiltip’) and sample probability (reflecting the proportion of sampled taxa compared to actual diversity) (Table 1). The estimates inferred with the uniform-tree priors (Fig. 3, red 95% HPD for Coccomorpha and Neococcoidea; Fig. S2), resulted in significantly older estimates for Coccomorpha origin to 270 Ma. Figure 5 summarizes the age estimates for the split between Aphidomorpha and Coccomorpha, Coccomorpha and Neococcoidea lineages, obtained from different tip-dating analyses detailed in Table 1. The other tip-dating analyses did not substantially change the age estimates, but the 95% HPD range varied. Notably, tip priors with a uniform distribution resulted in much wider 95% HPD, especially for the root and Coccomorpha nodes (Fig. 4B and Fig. S3).

Third, we tested the two options for sample diversity available in the tip-dating analysis in MrBayes. Diversity sampling was used in combination with the FBD method and assumes that extant taxa are sampled to maximize diversity and that fossils are sampled randomly, which is suitable for higher-level diversity^41^. On the other hand, the ‘fossiltip’ parameter in the FBD model assumes that extant taxa are sampled randomly and that extinct taxa are sampled with constant rate and the lineage is definitively extinct. Our dataset was assembled to sample as many Recent scale insect families as possible, while the fossils are more likely to be a result of random sampling. Even though the diversified sampling seems more appropriate to our case, we assessed whether using ‘fossiltip’ had a significant effect. Whether using a fixed or uniform tip prior, the diversified sampled analyses (5, TD-A and TD-B) resulted in slightly younger estimates, and significantly narrower confidence intervals in the case of fixed-tip priors.

Finally, comparing TD-B vs. TD-C, and TD-A vs. TD-D allows testing the effect of sample probability on the analysis. The proportion of sampled taxa compared to the currently known diversity corresponds to 0.01 (more than 8,000 Recent species). We also tried a sample probability of 0.005, which would assume that the estimated diversity is 4 times higher. Although the median age estimates did not change, the 95% HPD was wider in the case of sample probability set at 0.005 and fixed-tip age priors. Finally, the successive removal of node calibrations in the node-dating approach did not significantly alter the age estimates (Fig. S5) In general, the root-age prior distribution has an important impact on age estimates, especially at the deeper nodes of the phylogeny, and not applying it results in estimates that are younger than even the fossil record (in this case the presence of Aphidomorpha in the Triassic).

## Discussion

Analytical methods now allow the incorporation of fossil taxa into divergence-time estimations, although the approach is recent and only rare examples are available so far^39^,^44^. To incorporate fossils into divergence-time estimates, morphological data are essential, and datasets that include the morphology of both Recent and extinct taxa have been assembled in only a few groups of insects. We anticipate that this study will encourage the integration of palaeontological and neontological data. Some limits regarding the current implementation need to be pointed out, however. MrBayes’ first implementation of the tip-dating approach, using a simple tree-uniform prior^39^ on empirical datasets, usually results in older nodes compared to the traditional node-dating approach^44^. More recently, the FBD model, combined with the “diversified sampling” assumption^41^ allows reducing the gap between age estimates and the known fossil record. Our study tested both tip-dating implementations and found similar trends: setting the tree clock prior to “uniform” resulted in older estimates for the origin of Coccomorpha and Neococcoidea, while the FBD prior under “diversified sampling” estimated even younger ages compared to the traditional node-dating approach. The FBD model estimates origin of the Coccomorpha in the beginning of the Triassic. We also examined how root priors influenced our age estimates. When removing the root node prior based on the oldest known Aphidomorpha, from the Triassic^45^, we found that the age estimates were significantly younger, pushing forward the origin of Coccomorpha to the Jurassic (Early for node-dating and Late for tip-dating). However, regardless of the difference in age estimates using the root-prior or not, the estimated age of the neococcoid lineage, older than 150 Ma, precedes the angiosperm radiation in the mid-Cretaceous.

The time of origin and evolution of scale insect main lineages remains controversial. Contemporary systematics of scale insects has focused on resolving family-level relationships^23^ and only intuitive frameworks for coccoids within a geological timescale has so far been provided^1^,^32^,^35^. Based on fossil inclusions alone, several archeococcoid lineages have not only survived but also acquired their contemporary morphology by the Early Cretaceous^28^,^35^, suggesting an origin of Coccomorpha during the Jurassic or earlier^18^. The estimates based on the tip-dating approach places the origin of Coccomorpha about 245 Ma, in the Early Triassic. Our hypothesized origin of scale insects pre-dates their first known fossil record by at least 100 million years, although our confidence intervals (95% HPD) indicate a minimum gap of 80 million years. This fossil gap has also been found in divergence-time studies of various groups (e.g., angiosperms^46^ and birds^47^). This will eventually need to be corroborated directly by Early Mesozoic fossils, though the minute size of scale insects precludes their remains, being resolved in rocks of even the finest grain. Fortunately, the life-like fidelity of scale insects in amber 135 to 20 Ma allows unambiguous phylogenetic interpretation of the younger fossils. Also, minute mites in the phytophagous group Eriophyoidea have been found in mid-Triassic amber^48^, so it is possible that sternorrhynchans may likewise be found. Interestingly, 97% of living Eriophyoidea feed on angiosperms – a situation analogous to that of coccoids – and the Triassic mites are very similar to modern groups, indicating that a gymnosperm diet well preceded an angiosperm one.

The view that the neococcoids are largely a Tertiary radiation^1^,^31^,^32^ needs to be revised. Four mid- to Early Cretaceous genera (*Rosahendersonia, Pennygullania, Palaeotupo* and *Inka*), albeit rare stem groups to Pseudococcidae and to all other neococcoids, reveal that neococcoids were significantly diversified by then. This is further supported by the finding that *Gilderius* and *Williamsicoccus* are Cretaceous sister groups to the mealybugs, family Pseudococcidae. Based on established neococcoid fossils in the Early Cretaceous and the estimates from this study, the neococcoid lineage originated ~235 Ma, with the split of some major lineages (e.g., Pseudococcidae, Coccidae and Diaspididae) occurring before the mid-Cretaceous. While the Cretaceous angiosperm radiations probably had little effect on the family-level diversification of coccoids, consistent with the establishment of Recent insect families during the Mezosoic^11^, it is possible that a rapid diversification within some families occurred, particularly for today’s most diverse families such as the Coccidae and Diaspididae.

Did this major group of angiosperm herbivores, the scale insects, diversify well before the angiosperm radiations? If we look at the fossil record only, the earliest definitive evidence of angiosperms in the fossil record is Early Cretaceous monosulcate, collumellar pollen from several continents, 133–139 Ma^49^. Earliest remains of tricolpate pollen, leaves with dichotomous venation, and flowers are slightly younger^46^. One explanation is that angiosperms, or stem-group angiosperms, may have been present in the early stages of coccoid evolution, but have just not been recognised as such or found. Also, there are several reports – but highly controversial – of Triassic monosulcate, columellar, angiosperm-like pollen from the mid-Triassic^50^, which have some but not all of the characteristics of crown-group angiosperm pollen. Other potential stem-groups to angiosperms in the Jurassic and Triassic include remains of leaves and wood, such as of the Triassic palm-like plant *Sanmiguelia*. Monosulcate pollen is very rare prior to and even within the earliest parts of the Cretaceous, so angiosperms and angiosperm stem-groups at this time were no doubt correspondingly rare; these would have been probably too sparse to sustain the diversification of Coccomorpha.

If we look at recent estimates of molecular divergence-times for the origin of angiosperms, most of them are not total-evidence analyses, and assess the origin of angiosperms into the Jurassic^51^ and even into the Triassic^52^,^53^, although these are considered controversial^54^. Regardless of the divergence time ages, all estimates agree with the direct fossil evidence that the major diversification of angiosperms – their radiation – occurred entirely within the Cretaceous^51^,^55^.

Did the neococcoid lineages (representing more than 90% of today’s scale insect species diversity) originate during the angiosperm radiation? Future studies using methods similar to what we used here will provide additional divergence-time estimates in angiosperms for comparison. So far, direct evidence from fossil coccoids as well as divergence-time estimates agree that diverse scale insect families, including many neococcoids, existed before the angiosperm radiation.

Therefore, it is our contention that from the Triassic to Early Cretaceous scale insects mostly fed on conifers and other gymnosperms that dominated the landscapes. This is supported by the occurrence of wingless female coccoids in Cretaceous ambers, all trees of which were definitely coniferous. The majority of Coccomorpha then shifted to angiosperms when these plants underwent a massive radiation ca. 115-80 Ma, and later. This mid-Cretaceous radiation is unequivocal, based on abundant well-preserved pollen, wood, leaves, and flowers^46^,^56^. Some diverse modern families of Coccomorpha probably originated during this time, such as Diaspididae and Coccidae, but the huge grade of lineages basal to these families diverged well before this.

A shift of the coccoids from gymnosperms onto angiosperms in the mid- to Late Cretaceous would reflect that this major group of herbivores probably did not initially co-speciate with angiosperms. An emerging view^57^ probably best explains the diversification of angiosperm herbivores: the physical and chemical diversity of angiosperm hosts must increase the heterogeneity of insect environments, particularly in marginal areas, and this facilitates population divergence and speciation of the insect herbivores.

## Methods

### Taxon sampling and data

In this study, we analysed two datasets: (i) A Recent-only taxon sampling consisted of 78 terminals and representing 27 Coccomorpha families as well as five Aphidomorpha outgroups (three Aphididae and two Adelgidae); (ii) A matrix of Recent + fossil taxa included the 78 Recent and 43 fossil terminals. The second dataset represents 44 of the 52 currently recognised families (16 of the 19 extinct families, and 28 of the 33 extant families). Details for each taxon with their classification and sampled fossil species with their amber deposits are provided in Tables S2 and S4. We used for the morphological matrix the dataset from Vea and Grimaldi^28^, which consisted of 174 characters (124 adult male, 50 adult female) scored for 112 taxa. The list of morphological characters is detailed in Vea and Grimaldi^28^.

Molecular data consisted of partial nuclear regions of 18S, 28S and EF-1a, either newly sequenced or downloaded from GenBank. For details on sequencing protocol, GenBank accession numbers, and data processing prior to analyses, see Supplementary text. The ingroup taxon sampling was defined to maximise family-level representation of the Coccomorpha and, to account for the availability of male morphological knowledge (as it is the only type of morphological characters available for extinct forms) as much as possible and the potential availability of molecular sequences. Therefore, we completed the matrix with additional Recent taxa: *Matsucoccus microcicatrices, Puto superbus, P. albicans, Eumargarodes laingi, Bambusaspis miliaris* (Asterolecaniidae), *Kuwania* sp., *Crypticerya genistae, Marchalina hellenica* (Marchalinidae), and *Phoenicococcus marlatti* (Phoenicococcidae). This addition enabled to increase the representation of Coccomorpha families to 28 extant and 16 extinct families among the 33 and 19 families recognised respectively.

Despite our efforts to cover the largest family-level diversity, as well as obtaining as much data completeness as possible, the final taxon sampling had a heterogeneous data coverage (Fig. S1). All fossil taxa had only male morphology coded, while 43 out of the 78 Recent taxa had all three types of data (male morphology, female morphology and at least one of the three molecular markers). The final aligned and combined matrix is available in GitHub (https://goo.gl/BEpMeB).

### Divergence-time analyses

All phylogenetic hypotheses and divergence time estimates were inferred using MrBayes 3.2.6^58^ and followed the Independent Gamma Rates (IGR) relaxed-clock model, shown to perform better for tip-dating analyses using simulated data^39^,^59^. We performed two main types of analyses for this study. With the Recent-only dataset, we used the node-dating approach (ND) and 12 age-priors were assigned (or node calibration), this including a node-prior set at the crown ingroup and one at the root (split between Aphidomorpha and Coccomorpha). The second type of analysis used the Recent + fossil dataset and followed the tip-dating approach (TD), with all fossil terminals being assigned an age prior (or tip calibration)^39^. To assess the effect of priors, we performed a series of analyses by changing the following priors: assigning the root age prior with an offset exponential or log-normal distribution, or without root prior (ND and TD); removing successively each node age prior (ND); assigning the tip prior as fixed or with a uniform distribution (TD, cf. Table S4); setting the branch length prior as uniform^39^ or using the recently developed Fossilized Birth-Death model (FBD)^40^ implemented in MrBayes 3.2.6^41^ (TD); when using the FBD model, setting the sample strategy with extant taxa randomly sampled and extinct taxa sampled with constant rate until sampling (default option fossiltip), or with extant taxa sampled to maximise diversity and fossil taxa sampled randomly (option diversity^41^); finally, varying the sample probability to 0.01 or 0.05. Four replicates of 20 to 80 million generations were run with initial temperature of 0.1. We considered that convergence was achieved when the average standard deviation of split frequency was below 0.05. All trees were summarised using the command sumt in MrBayes with the option contype = allcompat. We chose to summarise the trees using all compatibility method because of the amount of missing data (fossils and a number of Recent terminals do not have molecular sequences) that tend to reduce significantly the posterior probabilities. For calibration information (node and tip priors) and further details on divergence time analyses, refer to Supplementary Text. All estimated ages presented in the text are the estimated median age followed by the 95% highest posterior density (HPD) in brackets. Finally, we used the cophyloplot function in R Ape package^60^ to compare the relationships retrieved from both approaches.

Combined and aligned matrices: GitHub (https://goo.gl/BEpMeB).

Phylogenetic trees: TreeBASE (http://purl.org/phylo/treebase/phylows/study/TB2:S17496).

## Additional Information

Accession codes: Genbank accessions: KT199022-KT199097. 

**How to cite this article**: Vea, I. M. and Grimaldi, D. A. Putting scales into evolutionary time: the divergence of major scale insect lineages (Hemiptera) predates the radiation of modern angiosperm hosts. *Sci. Rep.* **6**, 23487; doi: 10.1038/srep23487 (2016).

## Supplementary Material

Supplementary Information

## Figures and Tables

**Figure 1:**
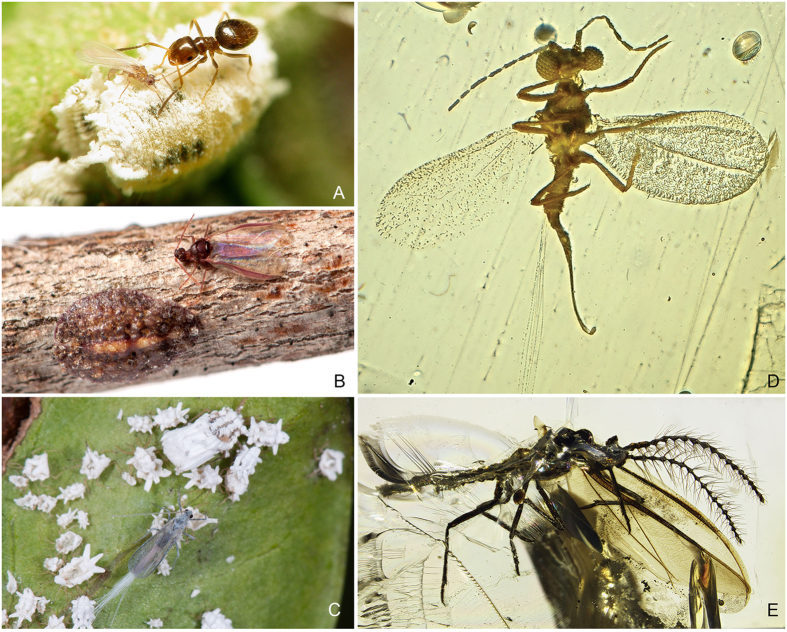
Representatives of Coccoidea showing extreme sexual dimorphism: neococcoid families. (**A**) Pseudococcidae adult female (center) and male (small on female, left of ant) *Phenacoccus* sp., credit: Sergio Jansen González, (**B**) Coccidae adult female (left) and male (right) *Pulvinaria acericola* (Walsh & Riley), credit: Matt Bertone, (**C**) “archeococcoid” family: Ortheziidae adult female (top) and male (bottom) *Praelongorthezia praelonga* (Douglas), credit: Mark Yokoyama, (**D**) inclusion of *Kozarius perpetuus* (Vea & Grimaldi) in mid-Cretaceous Burmese amber (~100 Ma), (**E**) inclusion of *Hodgsonicoccus patefactus* (Vea & Grimaldi) in Lebanese amber (~130 Ma).

**Table 1:** Details of tip-dating (TD) analyses performed in the present study.

	**Tip prior distribution**	**Sample strategy**	**Sample probability**	**Root prior distribution**
TD-A	fixed	diversity	0.01	offset exponential
TD-B	uniform	diversity	0.01	offset exponential
TD-C	uniform	diversity	0.005	offset exponential
TD-D	fixed	diversity	0.005	offset exponential
TD-E	uniform	fossiltip	0.01	offset exponential
TD-F	uniform	diversity	0.01	log-normal
TD-G	fixed	fossiltip	0.01	offset exponential
TD-H	fixed	diversity	0.01	no root
TD-I	fixed	diversity	0.01	log-normal
