# Supplementary material for: Putting scales into evolutionary time: the divergence of major scale insect lineages (Hemiptera) predates the radiation of modern angiosperm hosts
Source: Sci Rep. 2016 Mar 22;6:23487. doi: 10.1038/srep23487 (PMC4802209; doi:10.1038/srep23487)
Supplement: Supplementary Information [file srep23487-s1.pdf]

Electronic Supplementary Materials

Putting scales into evolutionary time: the divergence of major scale insect lineages (Hemiptera) predates the radiation of modern angiosperm hosts

Isabelle M. Vea [1,2] and David A. Grimaldi [2]

[1] Richard Gilder Graduate School, American Museum of Natural History, Central Park West at 79th street, New York, NY 10024

[2] Division of Invertebrate Zoology, American Museum of Natural History, Central Park West at 79th street, New York, NY 10024

Additional Materials and Methods Section:

(a) Material, DNA isolation and sequencing

Specimens used for molecular sequencing were either preserved in 100%, 70% ethanol, or acetone. Total genomic DNA was extracted using a DNeasy tissue extraction kit (Qiagen, Valencia, CA, USA), following the manufacturer's instructions, except for the following: whole specimens were left in lysis buffer and proteinase K overnight, two elutions of 50 µL were obtained at the end of the extraction, and the cuticle of each specimen was retrieved from the extraction column for slide preparation and identification.

Three nuclear markers were selected based on previous molecular studies on Coccomorpha: partial 18S, two regions of 28S (D2-3 and D10) and a region of EF-1 alpha. Table S1 provides the list of primers used for the amplification of the four regions as well the references from which they were selected; Genbank accession numbers are available on Table S2. PCR amplifications were performed with a Mastercycler ep Gradient S (Eppendorf) and consisted of 25 µL reactions with Illustra Ready-To-Go PCR beads (GE Healthcare), 1 µL of each primer (x10 dilution) and 2 to 4 µm of DNA template, depending

on the quantity of DNA retrieved from the extraction. For 18S and 28S fragments, PCR conditions followed that of Hardy et al. [1]. The EF-1a fragment was amplified using two sets of primers covering two overlapping regions, which resulted in a fragment of ~ 1080 bp. Conditions for PCR follow that of [2,3]. PCR products were purified using AMPure magnetic beads (Agencourt) and cycle-sequenced with the BigDye 1.1 Terminator Reaction Mix (Applied Biosystems, Inc.) and the same set of primers as PCR reactions. This protocol allowed a faster cycle sequencing program and used less BigDye. Cycle sequenced products were purified using CleanSeq (Agencourt) or ethanol precipitation. Sequencing was performed using an ABI 3730xl DNA Analyzer. Sequences were then compiled and edited using Geneious 5.1.7 [4] and saved in separate Fasta files for further analyses with additional sequences retrieved from Genbank. Sequence alignments were performed using MAFFT [5] through the Geneious platform. Introns from EF-1a were removed after alignment and the gene was partitioned by codon position (1st and 2nd as one partition and 3rd as one partition). The best-fit model of nucleotide substitution for each of the three markers was assessed using jModeltest 2.1.4 [6] and the best models under the Bayesian Information Criterion (BIC) were: TIM3ef+I+G for 18S, TVM+I+G for both 28S regions combined, TPM2+I+G for the partition including the 1st and 2nd codon positions of EF-1a, and HKY+G for the partition including the 3rd codon position of EF-1a. As TIM3ef, TVM and TPM models are not implemented in MrBayes, we used the GTR model for these cases.

Table S1. List of primers for each genetic marker and reference used in this study

Region	Primer sequences	Reference
18S (2880-Br)	CTG GTT GAT CCT GCC AGT AG<br>CCG CGG CTG CTG GCA CCA GA	[7]
28S (D2-D3)<br>s3360-28b	GAG AGT TMA ASA GTA CGT GAA AC<br>TCG GAA GGA ACC AGC TAC TA	[2]
28S (D9-D10)	GTA GCC AAA TGC CTC GTC A<br>CAC AAT GA TAG GAA GAG CC	[8]
EF-1a (2103–2342c)	CAC ATY AAC ATT GTC GTS ATY GG<br>CTT GAT GAA ATC YCT GTG TCC	[9]
EF-1a (40.6F-52R)	ATY GAR AAR TTY GAR AAR GAR GC<br>CCD ATY TTR TAN ACR TCY TG	[10]

(b) Taxon sampling and data coverage

Our study used the morphological matrix from Vea and Grimaldi [11], which included 174 morphological characters of male and female and was coded for 69 Recent taxa and 43 extinct taxa. In addition to this sampling, we added nine Recent for in which molecular data were available. These are: *Matsucoccus microcicatrices*, *Puto superbus, P. albicans, Eumargarodes laingi, Bambusaspis miliaris, Kuwania* sp., *Crypticerya genistae, Marchalina hellenica, and Phoenicococcus marlatti.*

Our final taxon sampling had varied data coverage as presented in Figure S1. All fossil taxa had only male morphology coded, while 43 out of the 78 Recent taxa had all three types of

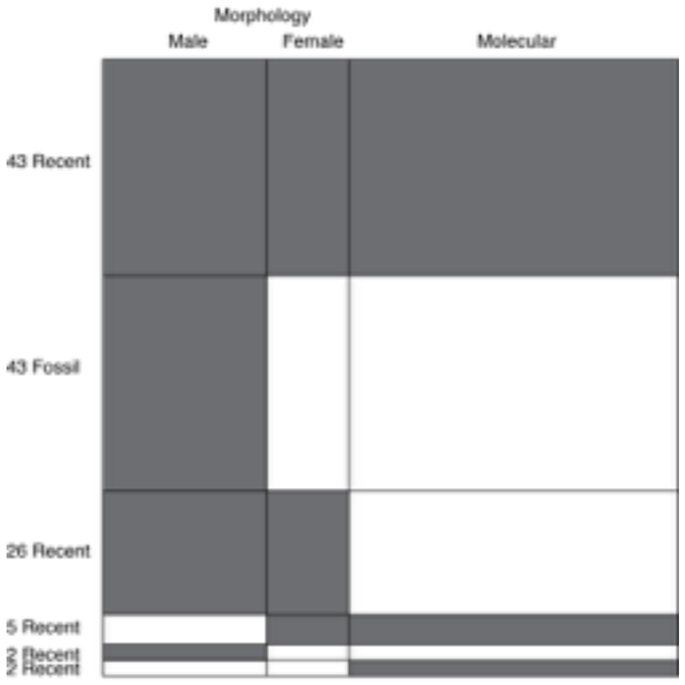

Figure S1: Diagram of data coverage for the combined molecular and morphological dataset. Grey areas represent coded data, white areas are missing data.

data (male morphology, female morphology and molecular sequence).

The combined matrices of Recent only and Recent and fossil sets are available as nexus files in GitHub. The original matrix from Vea and Grimaldi [11] is available in Morphobank under Project 1013. The final taxon sampling with GenBank accession numbers for molecular sequences is available in Table S2.

Table S2. Recent taxon sampling including GenBank accession numbers for molecular sequences (New accession numbers from this study are in bold), and percentage of scored morphological characters for male and female (Taxa with newly coded morphological characters not in Vea and Grimaldi (2015) with a *).

	Family	Species for morphology	Coded char. Male (%)	Coded char. Female (%)	Species for molecular data	18S	28S D2	28S D10	EF-1a	Material Depository or collected locality
1	Aclerdidae	*Aclerda arundinariae*	98.39	100	*Aclerda sp.*	AY426060	---	AY427412	---	*NA*
2	Adelgidae	*Adelges tsugae*	89.52	20	*A.tsugae*	**KT199045**	---	---	**KT199096**	Black Rock Forest, NY, USA
3	Adelgidae	*Pineus strobi*	90.32	100	*P.strobi*	---	---	---	EF073262	*NA*
4	Aphididae	*Acyrthosiphon pisum*	96.77	94	*A.pisum*	**KT199046**	---	---	**KT199097**	Crete, Greece
5	Aphididae	*Eucallipterus tiliae*	96.77	6	*E.tiliae*	---	---	---	---	*NA*
6	Aphididae	*Rhopalosiphum padi*	64.52	100	*R.padi*	U27825	---	---	---	*NA*
*7	Asterolecaniidae	---	---	---	*Bambusaspis.miliaris*	---	GU998966	---	---	*NA*
8	Beesoniidae	*Beesonia dipterocarpi*	90.32	100	*B.napiformis*	AY795511	---	---	---	*NA*
9	Besesoniidae	*Gallacoccus secundus*	88.71	100	*G.heckrothi*	AY795512	---	---	---	*NA*
10	Callipappidae	*Callipappus* sp.	100	96	---	---	---	---	---	*NA*
11	Cerococcidae	*Cerococcus artemisiae*	94.35	94	---	---	---	---	---	*NA*
12	Coccidae	*Ceroplastes japonicus*	100	98	*Ceroplastes sp.*	**KT199043**	**KT199065**	---	**KT199094**	AMNH-AMCC 200571
13	Coccidae	*Coccus hesperidum*	99.19	100	*C.hesperidum*	JX556916	JX645350	---	GU349853	*NA*
14	Coccidae	*Eulecanium tiliae*	100	96	*Eulecanium kunoense*	**KT199036**	---	---	**KT199091**	California State Collection of Arthropods
15	Coelostomidiidae	*Coelostomidia pilosa*	99.19	98	*C.pilosa*	**KT199042**	**KT199064**	---	---	NZAC02014796
16	Coelostomidiidae	*Coelostomidia wairoensis*	99.19	100	*C.wairoensis*	**KT199041**	**KT199063**	**KT199083**	---	NZAC02014840

	Family	Species for morphology	Coded char. Male (%)	Coded char. Female (%)	Species for molecular data	18S	28S D2	28S D10	EF-1a	Material Depository or collected locality
17	Coelostomidiidae	*Ultracoelostoma assimile*	98.39	98	---	---	---	---	---	*NA*
18	Conchaspididae	*Conchaspis lata*	98.39	100	---	---	---	---	---	*NA*
19	Dactylopiidae	*Dactylopius coccus*	100	100	*D.coccus*	---	---	---	---	*NA*
20	Dactylopiidae	*Dactylopius confusus*	99.19	100	*D.confusus*	**KT199039**	**KT199061**	**KT199081**	**KT199093**	AMNH-AMCC 200598
21	Diaspididae	*Aonidiella aurantii*	100	100	*A.aurantii*	U06475	JQ434503	---	---	*NA*
22	Diaspididae	*Chiocaspis salicis*	100	100	*C.salicis*	---	GU349105	---	---	*NA*
23	Diaspididae	*Parlatoria oleae*	99.19	100	*P.oleae*	---	GQ325522	---	GQ403835	*NA*
24	Eriococcidae	*Eriococcus coccineus*	100	100	*E.coccineus*	AY795336	EU746831	---	AY795503	*NA*
25	Eriococcidae	*Ovaticoccus agavium*	100	100	*O.agavium*	**KT199025**	---	**KT199068**	**KT199086**	California State Collection of Arthropods
26	Eriococcidae	*Tanyscelis verrucula*	87.9	98	*T.verrucula*	EU746803	EU746839	---	EU746899	*NA*
27	Eriococidae	*Eriococcus buxi*	100	98	*E.buxi*	AY795513	---	---	AY795493	*NA*
28	Kermesidae	*Kermes quercus*	96.77	98	*K.quercus*	---	JX436145	---	---	*NA*
29	Kermesidae	*Kermes* sp.	94.35	0	---	---	---	---	---	*NA*
30	Kerriidae	*Tachardina aurantiaca*	100	100	---	---	---	---	---	*NA*
*31	Kuwaniidae	---	0	0	*Kuwania sp.*	**KT199032**	**KT199055**	**KT199074**	---	Tsukuba, Ibaraki, Japan
32	Kuwaniidae	*Neosteingelia texana*	100	100	*N.texana*	**KT199028**	**KT199052**	**KT199071**	---	AMNH-AMCC 200597
33	Lecanodiaspididae	*Lecanodiaspis baculifera*	91.13	100	*L.baculifera*	**KT199031**	**KT199054**	**KT199073**	**KT199089**	California State Collection of Arthropods

	Family	Species for morphology	Coded char. Male (%)	Coded char. Female (%)	Species for molecular data	18S	28S D2	28S D10	EF-1a	Material Depository or collected locality
*34	Marchalinidae	*Marchalina hellenica*	0	100	*M.hellenica*	**KT199030**	EU087818	---	**KT199088**	AMNH-AMCC 200564
35	Margarodidae	*Dimargarodes meridionalis*	97.58	98	---	---	---	---	---	*NA*
36	Margarodidae	*Dimargarodes tanganyicus*	98.39	98	---	---	---	---	---	*NA*
*37	Margarodidae	*Eumargarodes laingi*	0	98	*E.laingi*	**KT199035**	**KT199058**	**KT199078**	---	AMNH-AMCC 200602
38	Margarodidae	*Hetermargarodes americanus*	100	98	---	---	---	---	---	*NA*
39	Margarodidae	*Porphyrophora hamelii*	100	100	---	---	---	---	---	*NA*
*40	Matsucoccidae	---	---	---	*Matsucoccus microcicatrices*	KF053072	KF040554	KF040584	---	*NA*
41	Matsucoccidae	*Matsucoccus bisetosus*	81.45	100	---	---	---	---	---	*NA*
42	Matsucoccidae	*Matsucoccus feytaudi*	97.58	100	---	---	---	---	---	*NA*
43	Matsucoccidae	*Matsucoccus josephi*	100	100	---	---	---	---	---	*NA*
44	Matsucoccidae	*Matsucoccus matsumurae*	95.97	100	*Matsucoccus sp.*	**KT199029**	**KT199053**	**KT199072**	---	AMNH-AMCC 200591
*45	Monophlebidae	*Crypticerya genistae*	0	98	*C.genistae*	**KT199040**	**KT199062**	**KT199082**	---	AMNH-AMCC 200599
46	Monophlebidae	*Drosicha corpulenta*	98.39	100	*D.corpulenta*	**KT199038**	**KT199060**	**KT199080**	**KT199092**	Tsukuba, Ibaraki, Japan
47	Monophlebidae	*Drosicha dalbergiae*	99.19	100	---	---	---	---	---	*NA*
48	Monophlebidae	*Drosicha pinicola*	99.19	100	*D.pinicola*	**KT199037**	**KT199059**	**KT199079**	---	Tsukuba, Ibaraki, Japan
49	Monophlebidae	*Gigantococcus maximus*	98.39	100	*G.maximus*	EU087721	---	EU087835	---	*NA*

	Family	Species for morphology	Coded char. Male (%)	Coded char. Female (%)	Species for molecular data	18S	28S D2	28S D10	EF-1a	Material Depository or collected locality
50	Monophlebidae	*Icerya purchasi*	100	98	*I.purchasi*	**KT199034**	**KT199057**	**KT199077**	AY429408	AMNH-AMCC 200576
51	Monophlebidae	*Icerya seychellarum*	100	100	*I.seychellarum*	**KT199033**	---	**KT199076**	**KT199090**	Nakagamigun, Okinawa, Japan
52	Monophlebidae	*Palaeococcus fuscipennis*	97.58	98	---	---	---	---	---	*NA*
53	Ortheziidae	*Insignorthezia insignis*	96.77	100	*Insignorthezia sp.*	HQ893784	**KT199056**	**KT199075**	---	AMNH-AMCC 192453
54	Ortheziidae	*Newsteadia floccosa*	99.19	100	*N.floccosa*	**KT199027**	**KT199051**	**KT199070**	---	MNHN14795
55	Ortheziidae	*Orthezia urticae*	99.19	100	*Orthezia* sp.	**KT199026**	**KT199050**	**KT199069**	**KT199087**	AMNH-AMCC
56	Ortheziidae	*Praelongorthezia praelonga*	96.77	98	---	---	---	---	---	*NA*
57	Phenacoleachiidae	*Phenacoleachia species a*	99.19	100	---	---	---	---	---	*NA*
58	Phenacoleachiidae	*Phenacoleachia species b*	97.58	0	---	---	---	---	---	*NA*
*59	Phoenicococcidae	*Phoenicococcus marlatti*	0	100	*P.marlatti*	**KT199024**	**KT199049**	---	---	AMNH-AMCC 200592
60	Pityococcidae	*Pityococcus* sp.	99.19	100	---	---	---	---	---	*NA*
61	Pseudococcidae	*Antonina graminis (female)/ A. crawii (male)*	99.19	100	*A.graminis*	**KT199044**	**KT199060**	**KT199084**	**KT199095**	AMNH-AMCC 200595
62	Pseudococcidae	*Ceroputo pilosellae*	98.39	96	---	---	---	---	---	*NA*
63	Pseudococcidae	*Coccidohystrix insolitus*	96.77	100	---	---	---	---	---	*NA*
64	Pseudococcidae	*Dysmicoccus grassii*	98.39	100	*D.alazon*	JF965398	JQ651254	JF965409	AY427279	*NA*
65	Pseudococcidae	*Ferrisia virgata*	99.19	98	*F.virgata*	AY426079	AY179468	AY427373	---	*NA*

	Family	Species for morphology	Coded char. Male (%)	Coded char. Female (%)	Species for molecular data	18S	28S D2	28S D10	EF-1a	Material Depository or collected locality
66	Pseudococcidae	*Planococcus citri*	100	100	*P.citri*	JF965405	---	AY427375	AY427264	*NA*
67	Pseudococcidae	*Pseudococcus longispinus*	98.39	199	*P.longispinus*	**KT199023**	**KT199048**	AY427400	**KT199085**	New York, NY, USA
68	Pseudococcidae	*Pseudococcus maritimus*	76.61	100	*P.maritimus*	AY426043	AY427312	AY427384	AY427265	*NA*
*69	Putoidae	---	---	---	*P.albicans*	AY426051	AY427838	AY427408	AY427301	*NA*
70	Putoidae	*Puto kozstarabi*	98.39	100	---	---	---	---	---	*NA*
71	Putoidae	*Puto mexicanus*	95.97	100	---	---	---	---	---	*NA*
*72	Putoidae	*Puto superbus*	0	100	*P.superbus*	**KT199022**	**KT199047**	**KT199067**	---	Katowice Forest Park, Poland
73	Putoidae	*Puto yuccae*	100	100	*P.yuccae*	AY426052	AY427339	---	---	*NA*
74	Steingeliidae	*Steingelia gorodetskia*	99.19	100	---	---	---	---	---	*NA*
75	Stictococcidae	*Stictococcus vayssievei*	88.71	98	*S.sjostedti*	AY795509	---	---	AY795494	*NA*
76	Stigmacoccidae	*Stigmacoccus asper*	99.19	98	---	---	---	---	---	*NA*
77	Xylococcidae	*Xylococculus betulae*	99.19	100	---	---	---	---	---	*NA*
78	Xylococcidae	*Xylococcus japonicus*	99.19	98	---	---	---	---	---	*NA*

(c) Divergence time analyses

All analyses were run in MrBayes 3.2.6 through the Cipres Science Gateway Portal [12]. Four replicates of 10 to 80 million generations were run at an initial temperature of 0.2 for preliminary analyses (non-clock and strict-clock) and 0.1 for the divergence time estimate analyses. Convergence was considered achieved when the average standard deviation of split frequency was below 0.05. All trees were summarized using the command sumt in MrBayes with the option contype=allcompat. We chose to summarize the trees using all compatibility method because of the amount of missing data (fossils and a number of Recent terminals do not have molecular sequences) that tend to reduce significantly the posterior probabilities.

Preliminary analyses

A non-clock and strict-clock analyses were first performed with Coccoidea as a topology constraint in order to define prior values for the IGR model and the clock rate. For details on command lines, see the codebook available publicly in Github (https://github.com/zourloubidou/Coccomorpha-divergence-time.md).

IGR model

The non-clock and strict-clock topologies were used as inputs to determine the prior distribution values for the IGR model and clock rate using the R script from Ronquist et al. [13].

Mean TH	Variance	sigma	age of root in million years	#substitution per million year	mu	Median variance branch length	parameter for igrvar
0.560715	0.002692	0.051884487	240	0.002336313	-6.060527447	0.03899406	25.64493156

Divergence-time estimate analyses

Both node-dating and tip-dating analyses used a relaxed clock following the IGR model from Ronquist et al. [13]. For the node-dating analyses, we used the Recent only dataset (fossil terminals were defined in a variable using the command line “taxset fossils =” and excluded from prior to analysis using the command line “remove fossils;”), for which node age priors were set for the root, the ingroup and 10 families following an off set exponential distribution. All details on node calibration can be found in Table S3. For the tip-dating method, we used the Recent+fossil dataset and performed fixed prior ages on all fossil terminals (Table S4 for details on ages) as well as an offset exponential distribution for the root prior. In order to assess if any of node prior and the root prior distribution (either in the node-dating analysis or the tip-dating where a prior distribution was defined for the root) could influence the age estimates in MrBayes, we performed various analyses with both node-dating and tip-dating approaches as follow:

- Node-dating method: 10 additional node-dating analyses were performed using all node priors and excluding one node prior at a time, as well as one analysis excluding the root prior, and one analysis changing the distribution of the root to lognormal (mean=240, SD=10).
- Tip-dating method: two additional analyses were performed with for the first one excluding the root prior, and the second changing the root prior distribution to lognormal (mean=240, SD=10).

All topologies with age estimates resulting from these analyses are available in TreeBASE (temporary link for reviewers: http://purl.org/phylo/treebase/phylows/study/TB2:S17496?x-access-code=4cff04a26ac567ac00b91ec3451e380d&format=html)

Table S3. Node calibration priors used for the node-calibrated analyses

Node	Family	Age prior	Fossil taxon	Reference	Remarks
1	root	offsetexp(240,250)	*Vosegus triassicus* Szwedo & Nel	[14]	The minimum age of the root was defined based on the wing impression of the oldest Aphidomorpha described in a rock deposit from the Vosges, France (early Anisian, Middle Triassic,~240 Ma).
2	Coccomorpha	offsetexp(140,240)	*Eomatsucoccus casei* Koteja	[15]	This calibration is based on the oldest definitive Coccoidea, belonging to the extant family Matsucoccidae. The minimum age was defined at 140 Ma as MrBayes did not allow to perform the analysis when the minimum age was set at the same age as a more inclusive node.
3	Coccidae	offsetexp(98, 110)	*Rosahendersonia prisca* Vea & Grimaldi	[11]	*Rosahendersonia prisca* was described from Burmese amber and is the oldest record of Coccidae. *R. prisca* is in the total evidence analysis taxon sampling.
4	Diaspididae	offsetexp(50, 100)	*Normarkicoccus cambayae*	[11]	*Normarkicoccus* sp. was described in the Diaspididae based on multiple characters, despite the presence of some plesiomorphic features such as the absence of a long penial sheath. This is the oldest Diaspididae fossil record from Cambay amber. *Normarkicoccus cambayae* is in the total evidence analysis taxon sampling.
5	Kermesidae	offsetexp(45, 100)	*Sucinikermes kulickae* Koteja	[16]	*Sucinikermes* was described in Baltic amber based on an instar nymph and was assigned to the Kermesidae based on known living Kermesidae instars. *Sucinikermes kulickae* is not the total evidence analysis taxon sampling.
6	Kuwaniidae	offsetexp(45, 100)	*Hoffeinsia foldi* Koteja	[17]	*Hoffeinsia* sp. was described in Baltic amber. *Hoffeinsia* is not in the total evidence analysis taxon sampling.
7	Margarodidae	offsetexp(50, 100)	*Heteromargarodes hukamsinghi* Vea & Grimaldi	[11]	*Heteromargarodes* is a living genus, for which the only fossil species of the family was described in Cambay amber. *Heteromargarodes hukamsinghi* is in the total evidence analysis taxon sampling.

8	Matsucoccidae	offsetexp(135,140)	*Eomatsucoccus casei* Koteja	[15]	Eomatsucoccus casei is the oldest definitive Coccoidea described in the fossil record. Is in the total evidence analysis taxon sampling.
9	Ortheziidae	offsetexp(135,140)	*Cretorthezia hammanaica* Koteja & Azar	[18]	*Crethorthezia hammanaica* was described in Lebanese amber based on a macropterous male. C. hammanaica is in the total evidence analysis taxon sampling.
10	Pseudococcidae	offsetexp(135,140)	*Williamsicoccus megalops* Vea & Grimaldi	[11]	*Williamsicoccus* was described in Lebanese amber based on a macropterous male. *Williamsicoccus megalops* is in the total evidence analysis taxon sampling.
11	Putoidae	offsetexp(45, 100)	Undescribed *Puto* sp. in Baltic amber	Koteja's amber collection, Silesia University	Definitive *Puto* males are found in Baltic ambers but yet to be described.
12	Xylococcidae	offsetexp(135,140)	*Baisococcus victoriae* Koteja	[19]	*Baisococcus victoriae* was described from a rock impression of a macropterous male and was subsequently classified in the Xylococcidae family by Koteja (2000). However, based on the state of conservation and the limited number of characters observable on the specimen, this taxon was not used in the total evidence analysis.

Table S4. Extinct taxon sampling with age priors used in the total evidence divergence time analyses and % of coded characters. Fossil deposits abbreviations: Al: Alaska; Bu: Burmese, Le: Lebanese, Ba: Baltic; Bi: Bitterfield; Ca: Canada; Cb: Cambay, Ch: Charentes; NJ: New Jersey; Ta: Taimyr.

	Family	Taxon	Fossil deposit	Fixed age priors	Age prior with uniform distribution	% of coded characters
1	Albicoccidae	*Albicoccus dimai* Koteja	Bu	98	98; 99.5	35.63
2	Apticoccidae	*Apticoccus minutus* Koteja & Azar	Le	135	120;135	45.98
3	Apticoccidae	*Apticoccus longitenuis* Koteja & Azar	Le	135	120;135	43.68
4	Apticoccidae	*Apticoccus fortis* Koteja & Azar	Le	135	120;135	45.4
5	Arnoldidae	*Arnoldus capitatus* Koteja	Ba	45	43;48.5	43.68
6	Burmacoccidae	*Burmacoccus danyi* Koteja	Bu	98	98; 99.5	39.08
7	Coccidae	*Rosahendersonia prisca* Vea & Grimaldi	Bu	98	98; 99.5	44.83
8	Diaspididae	*Normarkicoccus cambayae* Vea & Grimaldi	Cb	50	50;52	38.5
9	Electrococcidae	*Electrococcus canadensis* Beardsley	Ca	100	95;105	48.8
10	Electrococcidae	*Turonicoccus beardsleyi* Koteja	NJ	92	88;94	32.18
11	Eriococcidae	*Kuenowicoccus pietrzeniukae* Koteja	Ba	45	43;48.5	47.7
12	Grimaldiellidae	*Grimaldiella gregaria* Koteja	NJ	92	88;94	56.9
13	Grohnidae	*Grohnus eichmanni* Koteja	Ba	45	43;48.5	47.7
14	Hodgsonicoccidae	*Hodgsonicoccus patefactus* Vea & Grimaldi	Le	135	120;135	51.15
15	Incertae sedis	*Marmyan barbarae* Koteja	Bu	98	98; 99.5	29.89
16	Incertae sedis	*Pedicellicoccus marginatus* Vea & Grimaldi	Bu	98	98; 99.5	43.68
17	Incertae sedis	*Undescribed fossil from French amber*	Ch	100	95;100	28.16
18	Incertae sedis	*Magnilens glaesaria* Vea & Grimaldi	Bu	98	98; 99.5	41.38
19	Incertae sedis	*Alacrena peculiaris* Vea & Grimaldi	Bu	98	98; 99.5	41.38
20	Incertae sedis	*Xiphos vani* Vea & Grimaldi	Le	135	120;135	51.77
21	Inkaidae	*Inka minuta* Koteja	Ta	85	80;90	57.47
22	Jersicoccidae	*Jersicoccus kurthi* Koteja	NJ	92	88;94	31.03
23	Kozariidae	*Kozarius achronus* Vea & Grimaldi	Bu	98	98; 99.5	54.6
24	Kozariidae	*Kozarius perpetuus* Vea & Grimaldi	Bu	98	98; 99.5	55.17

25	Kukaspididae	*Kukaspis usingeri* Koteja & Poinar	Al	100	95;100	47.12
26	Labiococcidae	*Solicoccus nascimbenei* Koteja	NJ	92	88;94	47.12
27	Lebanococcidae	*Lebanococcus longiventris* Koteja & Azar	Le	135	120;135	44.83
28	Margarodidae	*Heteromargarodes hukamsinghi* Vea & Grimaldi	Cb	50	50;52	41.95
29	Matsucoccidae	*Eomatsucoccus casei* Koteja	NJ	92	88;94	36.78
30	Ortheziidae	*Cretorthezia hammanaica* Koteja and Azar	Le	135	120;135	35.63
31	Ortheziidae	*Palaeonewsteadia huaniae* Koteja	Ba	45	43;48.5	51.72
32	Ortheziidae	*Protorthezia aurea* Koteja	Ba	45	43;48.5	44.83
33	Pennygullaniidae	*Pennygullania electrina* Koteja & Azar	Le	135	120;135	43.1
34	Pityococcidae	*Pityococcus moniliformalis* Vea & Grimaldi	Ba	45	43;48.5	64.37
35	Pseudococcidae	*Williamsicoccus megalops* Vea & Grimaldi	Le	135	120;135	43.68
36	Pseudococcidae	*Gilderius eukrinops* Vea & Grimaldi	Bu	98	98; 99.5	46.55
37	Putoidae	*Palaeotupo danielae* Koteja & Azar	Le	135	120;135	45.4
38	Serafinidae	*Serafinus acupiterus* Koteja	Ba	45	43;48.5	57.47
39	Steingeliidae	*Palaeosteingelia acrai* Koteja & Azar	Le	135	120;135	54.6
40	Steingeliidae	*Steingelia cretacea* Koteja	NJ	92	88;94	52.3
41	Weitschatidae	*Weistchatus stigmatus* Koteja	Ba	45	43;48.5	64.94
42	Weitschatidae	*Pseudoweitschatus audebertis* Vea & Grimaldi	Bu	98	98; 99.5	52.3
43	Xylococcidae	*Xylococcus grabenhorstii* Koteja	Bi	45	40;50	52.87

Ages and LTT plots

For a quick overview of age-estimate differences among the analyses, we obtained lineage through time (LTT) plots in R version 3.1.2 (2014-10-31) [20]. For all tip-dating analyses,

fossil terminals were pruned prior to obtained the LTT plots so that they could be comparable to the node-dated topologies. Age estimates for different analyses were also plotted for comparison using the ggplot2 package.

Command lines and packages used to obtain the plots are available in Github (https://github.com/zourloubidou/Coccomorpha-divergence-time/blob/master/Plots.md- for reviewer access, first login at http://www.github.com with username: Journalreviewer, password: Githubforreview2015).

Additional results section

(a) Dataset and output files

A README file is available in GitHub that summarizes all datasets and intermediate results made available to the reader.

(b) Tip-dating analysis using Ronquist et al., 2012

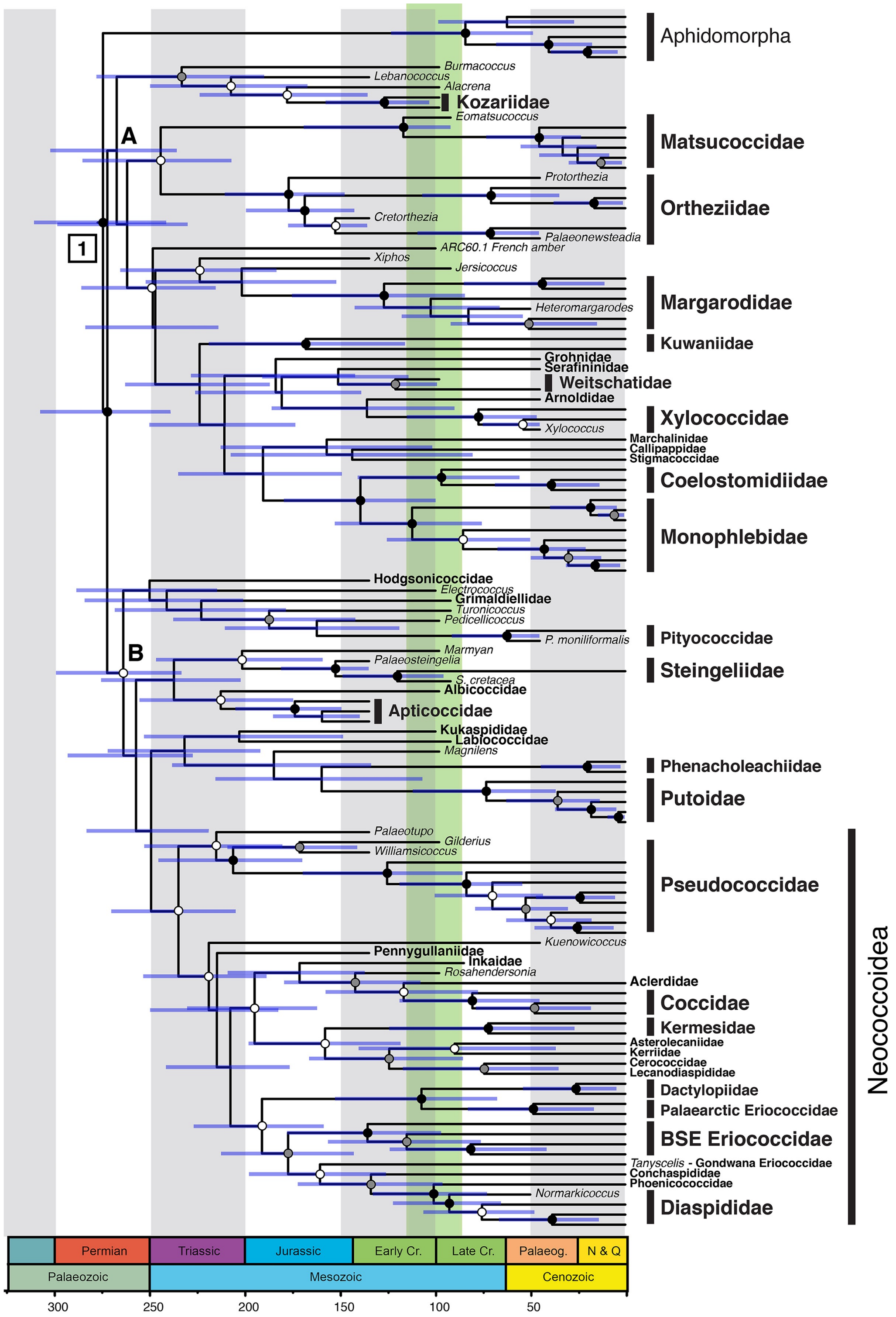

Figure S2: Divergence-time estimates based on a total-evidence approach (tip-dating) including Recent and fossil terminals, using MrBayes 3.2.3 from Ronquist et al., 2012 approach, all-compatibility summary. Posterior probability: black dot: 95-100% P; grey dot: 80-94%; white dots: 50-79%. Node number represents root node prior (Supplementary Table S3). Node letters are clades mentioned in the results. Green area: period of major angiosperm radiation.

(c) Posterior age plots and LTT Plots

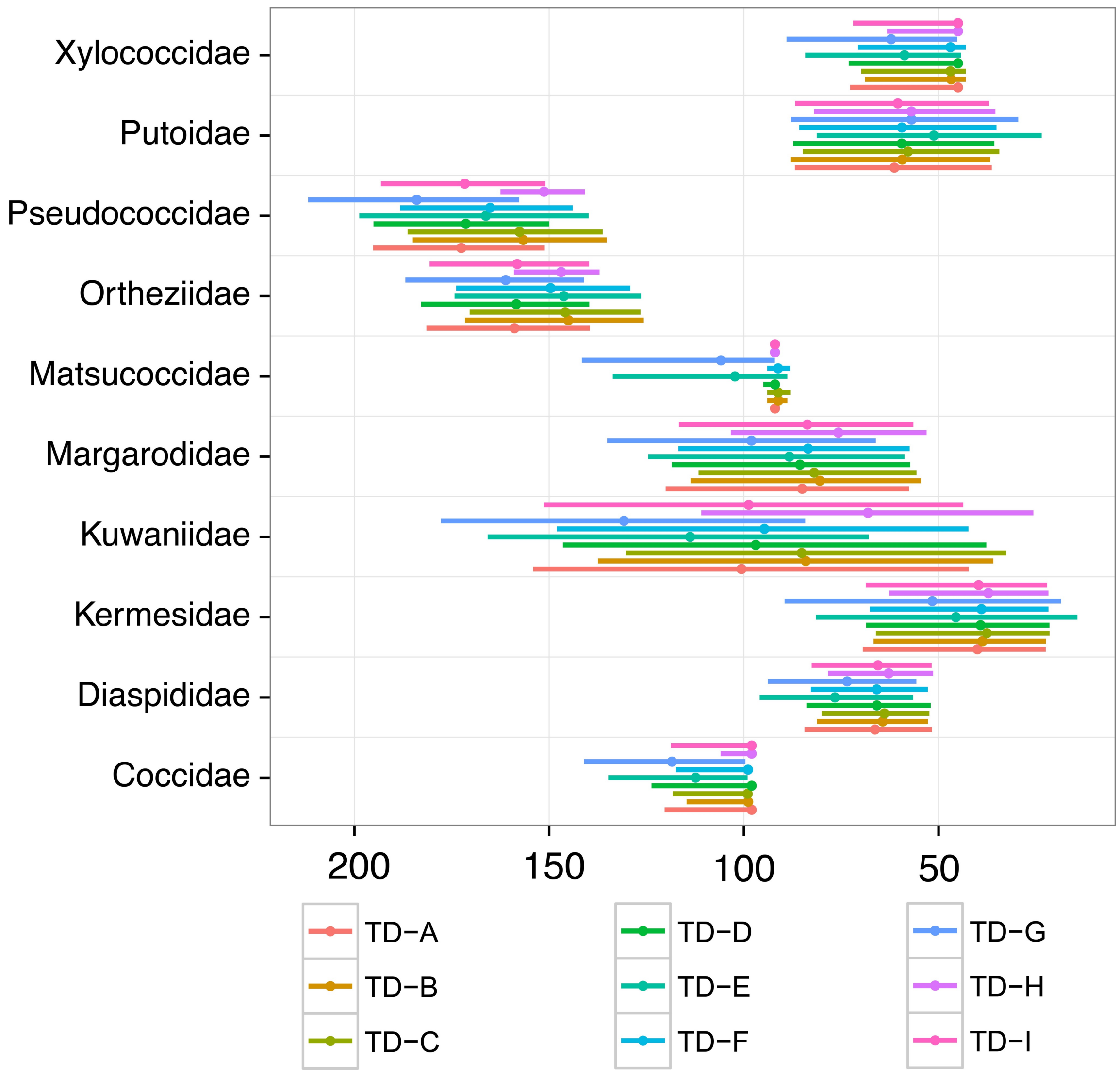

Figure S3: Comparison among tip-dating analyses of the posterior ages (median and 95 % HDP) for lineages with age priors used in the node-dating analysis. For the parameters used in each tip-dating analysis, refer to Table 1.

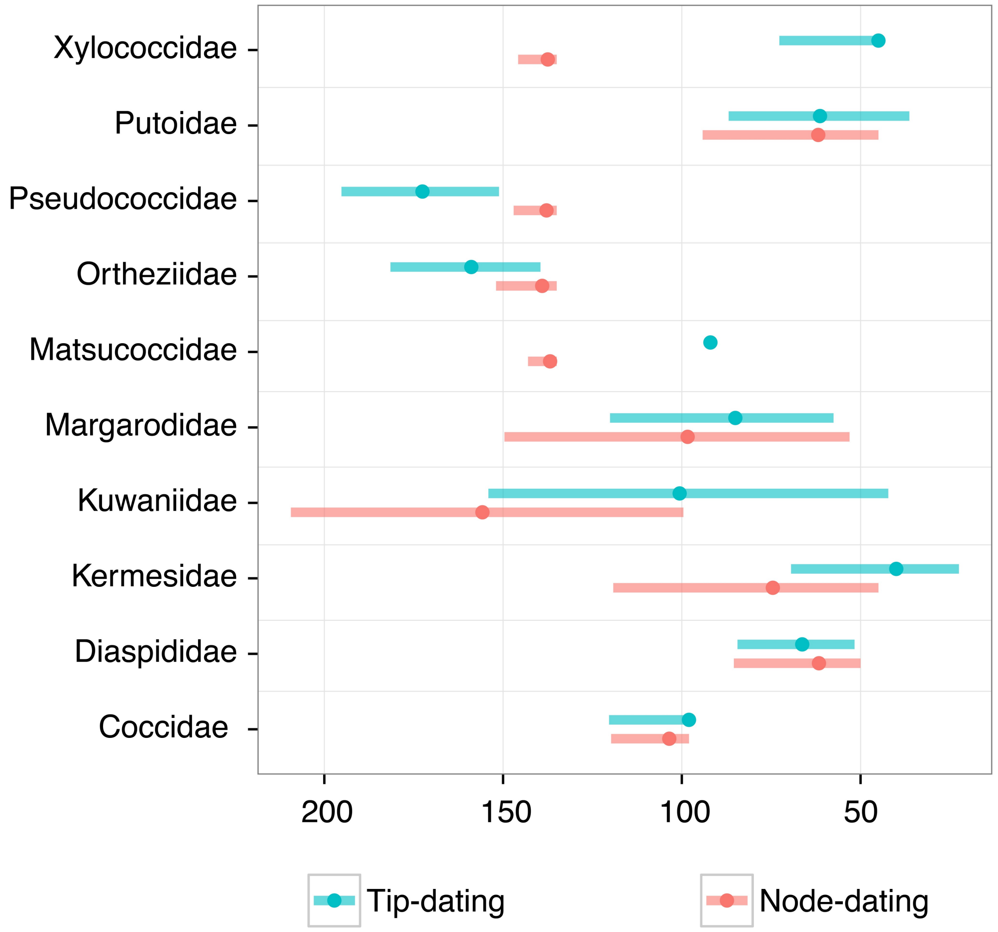

Figure S4: Comparison between node-dating and tip-dating approaches of the posterior ages (median) and 95% HDP for lineages with age priors used in the node-dating analysis.

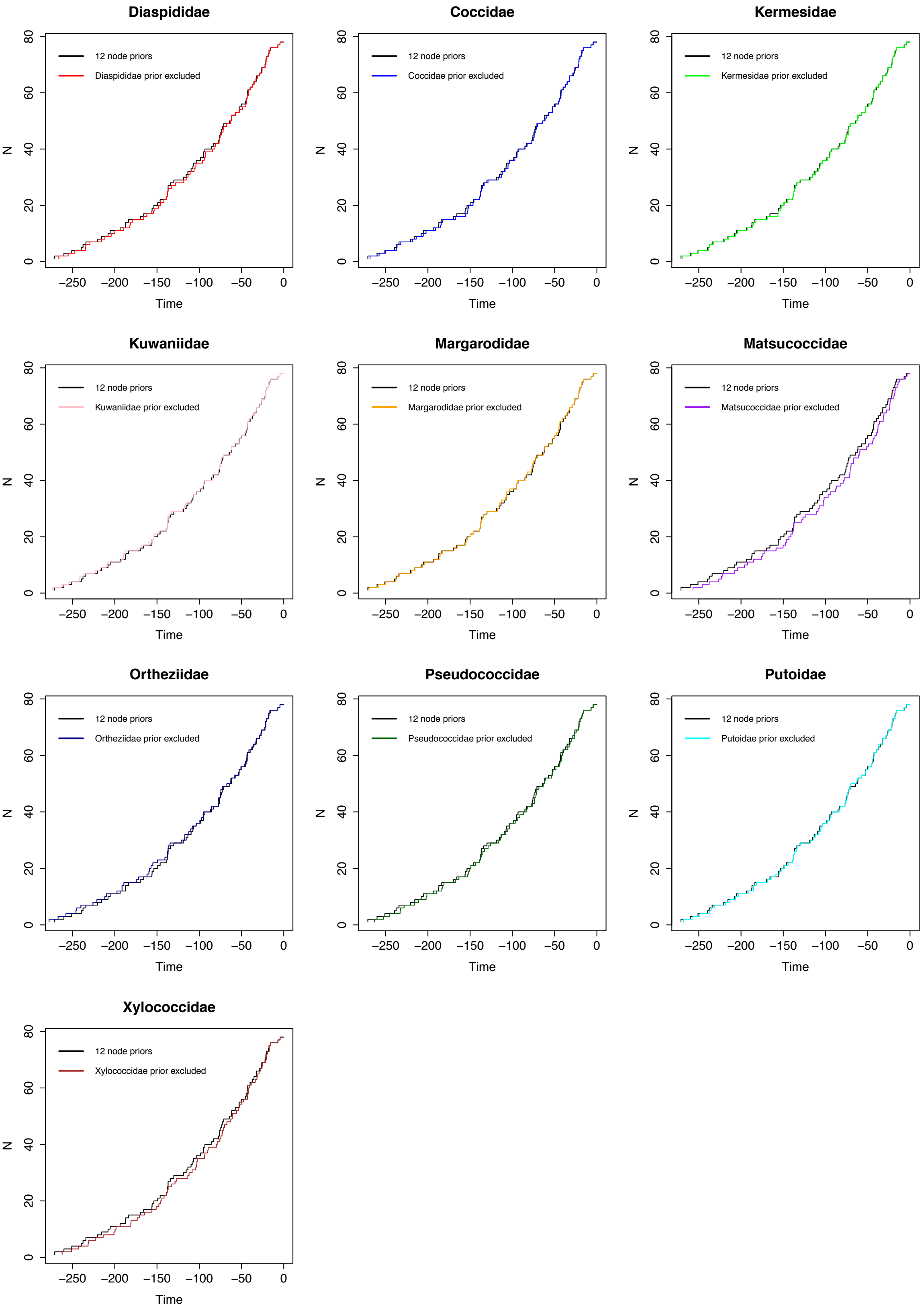

Figure S5: Effect on age estimates for the node-dating analyses when removing one of the node priors individually.

References

1. Hardy, N. B., Gullan, P. J., Henderson, R. C. & Cook, L. G. 2008 Relationships among felt scale insects (Hemiptera: Coccoidea: Eriococcidae) of southern beech, Nothofagus (Nothofagaceae), with the first descriptions of Australian species of the Nothofagus-feeding genus Madarococcus Hoy. *Invert. Systematics* **22**, 365. (doi: 10.1071/IS07032)

2. Morse, G. E. & Normark, B. B. 2005 A molecular phylogenetic study of armoured scale insects (Hemiptera: Diaspididae). *Systematic Entomology* **31**, 338–349. (doi: 10.1111/j.1365-3113.2005.00316.x)

3. Downie, D. A. & Gullan, P. J. 2005 Phylogenetic congruence of mealybugs and their primary endosymbionts. *Journal of Evolutionary Biology* **18**, 315–324. (doi:10.1111/j.1420-9101.2004.00834.x)

4. Kearse, M. et al. 2012 Geneious Basic: an integrated and extendable desktop software platform for the organization and analysis of sequence data. *Bioinformatics* **28**, 1647–1649. (doi:10.1093/bioinformatics/bts199)

5. Katoh, K., Misawa, K., Kuma, K. I. & Miyata, T. 2002 MAFFT: a novel method for rapid multiple sequence alignment based on fast Fourier transform. *Nucleic Acids Research* **30**, 3059–3066. (doi:10.1093/nar/gkf436)

6. Darriba, D., Taboada, G. L., Doallo, R. & Posada, D. 2012 jModelTest 2: more models, new heuristics and parallel computing. *Nat Meth* **9**, 772–772. (doi:10.1038/nmeth.2109)

7. Dohlen, Von, C. D. & Moran, N. A. 1995 Molecular phylogeny of the Homoptera: a paraphyletic taxon. *Journal of Molecular Evolution* **41**, 211–223. (doi:10.1007/bf00170675)

8. Dietrich, C. 2001 Phylogeny of the major lineages of Membracoidea (Insecta: Hemiptera: Cicadomorpha) based on 28S rDNA sequences. *Molecular Phylogenetics and Evolution* **18**, 293–305. (doi:10.1006/mpev.2000.0873)

9. Cho, S., Mitchell, A., Regier, J. C., Mitter, C., Poole, R. W., Friedlander, T. P. & Zhao, S. 1995 A highly conserved nuclear gene for low-level phylogenetics: elongation factor-1 alpha recovers morphology-based tree for heliothine moths. *Molecular Biology and Evolution* **12**, 650–656.

10. Regier & C, J. 2007 Protocols, Concepts, and Reagents for preparing DNA sequencing templates. 1–34.

11. Vea, I. M. & Grimaldi, D. A. 2015 Diverse new scale insects (Hemiptera: Coccoidea) in amber from the Cretaceous and Eocene with a phylogenetic framework for fossil Coccoidea. *American Museum Novitates*, 1–80. (doi:10.1206/3823.1)

12. Miller, M. A., Pfeiffer, W. & Schwartz, T. 2010 Creating the CIPRES Science Gateway for inference of large phylogenetic trees. 1–8.

13. Ronquist, F., Klopfstein, S., Vilhelmsen, L., Schulmeister, S., Murray, D. L. & Rasnitsyn, A. P. 2012 A total-evidence approach to dating with fossils, applied to the early radiation of the Hymenoptera. *Systematic Biology* **61**, 973–999. (doi:10.1093/sysbio/sys058)

14. Szwedo, J. & Nel, A. 2011 The Oldest Aphid Insect from the Middle Triassic of the Vosges, France. *Acta Palaeontologica Polonica* **56**, 757–766. (doi:10.4202/app.2010.0034)

15. Koteja, J. 2000 Scale insects (Homoptera, Coccinea) from upper cretaceous New Jersey amber. In *Studies on fossils in amber, with particular reference to the Cretaceous of New Jersey* (ed D. A. Grimaldi), pp. 147–229. Leiden: Backhuys Publishers.

16. Koteja, J. 1988 Sucinikermes kulickae gen. et sp. n. (Homoptera, Coccinea) from Baltic amber. *Polskie Pismo Entomologiczne* **58**, 525–535.

17. Koteja, J. 2008 Xylococcidae and related groups (Hemiptera: Coccinea) from Baltic amber. **49**, 19–56.

18. Koteja, J. & Azar, D. 2008 Scale insects from lower cretaceous amber of Lebanon (Hemiptera: Sternorrhyncha: Coccinea). *Alavesia* **2**, 133–167.

19. Koteja, J. 1989 Baisococcus victoriae gen. et sp. n.—a Lower Cretaceous coccid (Homoptera, Coccinea). *Acta Zoologica Cracoviensia* **32**, 93–106.

20. R Core Team 2014 R: A language and environment for statistical computing.
